# A new microspore embryogenesis system under low temperature which mimics zygotic embryogenesis initials, expresses auxin and efficiently regenerates doubled-haploid plants in *Brassica napus*

**DOI:** 10.1186/1471-2229-12-127

**Published:** 2012-08-02

**Authors:** Deepak Prem, María-Teresa Solís, Ivett Bárány, Héctor Rodríguez-Sanz, María C Risueño, Pilar S Testillano

**Affiliations:** 1 Plant Development and Nuclear Architecture, Centro de Investigaciones Biológicas, CIB-CSIC, Ramiro de Maeztu 9, 28040, Madrid, Spain

**Keywords:** Microspore embryogenesis, Zygotic-like microspore embryogenesis, Suspensor-like, *Brassica napus*, Doubled-haploid, Embryo germination, Plant regeneration, *In vitro* microspore culture, Auxin, IAA

## Abstract

**Background:**

Microspore embryogenesis represents a unique system of single cell reprogramming in plants wherein a highly specialized cell, the microspore, by specific stress treatment, switches its fate towards an embryogenesis pathway. In *Brassica napus,* a model species for this phenomenon, incubation of isolated microspores at 32°C is considered to be a pre-requisite for embryogenesis induction.

**Results:**

We have developed a new *in vitro* system at lower temperature (18°C) to efficiently induce microspore embryogenesis throughout two different developmental pathways: one involving the formation of suspensor-like structures (52.4%) and another producing multicellular embryos without suspensor (13.1%); additionally, a small proportion of non-responsive microspores followed a gametophytic-like development (34.4%) leading to mature pollen. The suspensor-like pathway followed at 18°C involved the establishment of asymmetric identities from the first microspore division and an early polarity leading to different cell fates, suspensor and embryo development, which were formed by cells with different organizations and endogenous auxin distribution, similar to zygotic embryogenesis. In addition, a new strategy for germination of microspore derived embryos was developed for achieving more than 90% conversion of embryos to plantlets, with a predominance of spontaneous doubled haploids plants.

**Conclusion:**

The present work reveals a novel mechanism for efficient microspore embryogenesis induction in *B. napus* using continuous low temperature treatment. Results indicated that low temperature applied for longer periods favours an embryogenesis pathway whose first division originates asymmetric cell identities, early polarity establishment and the formation of suspensor-like structures, mimicking zygotic embryogenesis. This new *in vitro* system provides a convenient tool to analyze *in situ* the mechanisms underlying different developmental pathways during the microspore reprogramming, breaking or not the cellular symmetry, the establishment of polarity and the developmental embryo patterning, which further produce mature embryos and plants.

## Background

In recent years, microspore culture for doubled haploids (DH) has become a routine biotechnological tool for value addition in crops and several successful DH protocols that can fast track traditional or modern plant breeding approaches have been described in *Brassicas*[1,2]. Rapeseed (*Brassica napus* L.), is considered as one of the model systems for studying microspore embryogenesis. Isolated microspore *in-vitro* cultures provide a unique opportunity to study the cellular events that mark reprogramming and totipotency at single cell level. In *B. napus* ever since the first report of embryogenesis induction in anther culture using a heat shock treatment [3], the use of a high temperature treatment i.e. 32°C for 3-21d, is accepted as the norm for developing microspore derived embryos both in cultured anthers and microspores (Charne and Beversdorf 1988, ( [4-8]). Apart from this, culture of isolated microspores of *B. napus* at 18°C has been proposed as an ideal system to study the gametophytic development *in-vitro*, wherein cultures with tricellular pollen (up to 90% viable) can be obtained after eight days of culture [9,10]. Recently Joosen and co-workers [11] have presented a comparative transcriptome and proteome analysis that identifies the two *in vitro* developmental pathways, embryogenic or gametophytic. They also reported a new *in vitro* system to obtain microspore embryos by a short 24 h incubation of isolated microspores to 32°C with the predominance of the suspensor throughout their development. These new developmental features in *B. napus* microspore embryogenesis have been further described in detail [12].

Previous results from anther/ microspore cultures of other horticultural herbaceous and woody species have also established that the application of a heat shock treatment (32-33°C) during the vacuolated phase efficiently induced microspores reprogramming to embryogenesis [13-18].

In plants, asymmetric cell division is essential in many developmental processes to maintain cell diversity and tissue patterns, as well as to generate new cell fates throughout development (reviewed in [19]). The breaking of symmetry is regulated by intrinsic and extrinsic factors determining the polarity in equally or unequally dividing cells [20]. The occurrence and relevance of asymmetric divisions have been studied in different plant developmental processes, as zygotic embryogenesis. Nevertheless, it has not been analyzed during microspore embryogenesis yet.

The cellular architecture specific changes that occur during the switch from gametophytic to embryogenic pathway following a heat shock treatment in *B. napus* have been reported earlier [10,21-25], however, in all these studies related to developmental fate of the microspore, most emphasis has been laid on the early stages of embryo development whereas the later fate of microspore embryos in terms of their germination, ploidy assessment and establishment of mature seed bearing plants has not been reported. In any alternate system of microspore embryos production, the efficiency in regenerating DH plants is essential to evaluate the physiological status of the microspore embryos produced by any pathway, and to assess the system as a breeding tool.

In the present work, a new *in vitro* system in which microspore embryogenesis was efficiently induced in *Brassica napus* at low temperature has been developed. Under 18°C treatment, the reprogrammed microspores followed two different developmental pathways to form embryos, the major pathway involving the formation of suspensor-like structures, and the minor pathway producing multicellular embryos without suspensor; additionally, a proportion of non-responsive microspores were able to follow a gametophytic-like development leading to mature pollen. Defined conditions for donor plant growth under low temperature that positively affect the efficiency of microspore embryogenesis were also determined. In addition, a new strategy for germination of microspore embryos was developed to achieve more than 90% conversion of embryos to plantlets. This new system provides an interesting *in vitro* tool for analyzing the cellular processes underlying the different developmental pathways taking place at early microspore reprogramming and embryogenesis leading to mature embryos and plants.

## Material and methods

### Plant material, donor plant growth conditions and microspore stage analysis

The donor plants of *Brassica napus* genotype Topas were grown in two distinct growth conditions. Seeds of donor plants were sown in 15cm plastic pots containing agropeat (Campo®, CAMPO Agricultura, Barcelona, Spain) and these were grown in a growth chamber maintained at 18°C/ 16 h photoperiod with light intensity (100 μmol m^-2^ s^-1^) and 60% relative humidity (RH) till crown initiation. Thereafter, the plants were shifted to two different growth conditions; (1) growth chamber (Sanyo MLR-351) maintained at 15°C day, 16 h photoperiod (190 μmol m^-2^ s^-1^),10°C night, 60 ± 5% RH) and (2) greenhouse at 18°C day/ night with ambient photoperiod and RH. Under both donor plant conditions, flower buds were collected within 2 h from start of photoperiod for microspore stage evaluation and subsequent isolation for culture. To establish the relationship between bud size and microspore stage of development, immediately after collection, the buds were graded into four bud sizes ranging from 3.0 to 3.9 mm (equal intervals of 0.2 mm) measured from the base to the tip of the bud. Sample preparation for fluorescence microscopy was done as per [26] with modifications. In order to derive a quantitative estimation, 10–12 buds of each bud size range were macerated in 3 ml of 10% (w/v) sucrose solution and the slurry was filtered through a 48μm nylon filter. The filtrate was centrifuged at 2000 rpm for 5 min at room temperature and after decanting the supernatant, the pelleted microspores were stained with 50 μL 4,6-diamidino-2-phenylindole (DAPI) solution [6 μg/ml in phosphate buffered saline (PBS)] and incubated for 2 h at room temperature. Slide preparations were observed under UV fluorescence using Zeiss Axioplan epifluorescence microscope equipped with a CCD camera. The cell counts were taken for different stages of microspores at 20 × magnification from 3–4 different fields from each slide. The three replicate percentage data for microspores from buds coming from the two donor growth conditions and four bud size categories was arcsine transformed and analysed by 2 × 4 factorial ANOVA [27]. Isolated microspores from different growth conditions and bud size categories were also fixed in paraformaldehyde and processed as per [18] for bioimaging analysis as detailed below. Microspore cultures were carried out for over two years and four passages of new donor plants in each growth condition.

### Microspore isolation and culture

The effect of temperature treatments on microspore embryogenesis for induction was tested using the donor plants grown above under two growth environment. The experiment was planned in a three replicate 2 × 3 factorial completely randomized design (CRD) wherein microspores were isolated from buds harvested from the two growth environments and subjected *in-vitro* to three different temperature conditions. Each replicate of the experiment consisted of at least two separate isolations events. The procedure described by Prem et al. [26] was followed for microspore isolations with minor modifications. Flower buds were graded according to size and predominance of vacuolated microspore stage. The selected buds were surface-sterilized in 5.0% (v/v) commercial bleach (5% active chlorine) for 20 min and then rinsed 6–7 times with sterile distilled water. Ten to 15 buds were crushed using a cold mortar and pestle in 5 ml of cold NLN-13 medium ([3]; Duchefa) containing 13% sucrose (w/v). The suspension was filtered through 48 μm nylon mesh and the filtrate collected in 15-ml falcon centrifuge tubes. The crushed buds were rinsed with 5 ml NLN-13 to make up the volume to 10 ml and the filtrate was then centrifuged at 1100 rpm for 5 min at 4°C. The pellet was re-suspended in 10 ml of cold NLN-13 and centrifuged as mentioned above. This process was repeated three times for washing of the microspores. The final pellet was suspended in the NLN-13, and the cell density was adjusted to 10,000 cells per ml. The cell suspension was then poured into 60-mm Petri dishes (2.5-3 ml per Petri dish). After isolation, cultures were subjected to three different temperature conditions in dark, namely, 32.0 ± 1°C, 18 ± 1°C and 25 ± 1°C (control) and checked every 2d under stereomicroscope till development of globular/ heart shaped embryos was observed. Thereafter, cultures were shifted to 25 ± 1°C on a gyratory shaker at 60 rpm until complete development and maturation of the embryos was observed, around 30 days for the system induced at 32°C, and 40 days for the new system at 18°C. Results were quantified in terms of number of embryos produced per Petri dish and quantitative analysis for embryogenic response was done by partitioning the overall experimental variation using 2 × 3 factorial ANOVA [27], while qualitative cell architecture analysis was carried out by bioimaging at various stages of microspore culture.

### Processing and staining techniques for microscopic analysis

The microspore cultures were monitored by bright field and phase contrast microscopy using an *in vivo* live cell imaging microscope (Leica AF6000 LX with Electron multiplier CCD camera Hamamatsu C9100-02). Samples from cultures were also analysed for observing cell division patterns using DAPI (6 μg/ml) squash preparations as described by Solis et al. [18]. Based on *in-vivo* live imaging and DAPI analysis, samples of cultured microspores, multicellular pro-embryos and embryo at different developmental stages from different experimental treatments, were fixed in 4% paraformaldehyde in PBS, overnight, at 4°C. They were embedded in gelatin and processed as per Solis et al. [18]. Selected samples were dehydrated in acetone series and embedded in Technovit 8100 resin (Kulzer, Germany) at 4°C. Some semi-thin sections (1 μm) were stained with toluidine blue (0.075% toluidine blue in water) for structural analysis [18]. After washing and drying, sections were mounted in Eukitt and observed under bright field. DAPI staining (6 μg/ml) was used for observing nuclear components under UV irradiation in squash preparations.

For detection of dead cells, Evan’s blue staining was directly performed on culture samples as reported previously [28]. All slide preparations were observed with a Zeiss Axioplan epifluorescence microscope equipped with a CCD camera.

### Immunofluorescence for auxin localization

Semithin Historesin sections were incubated with 5% bovine serum albumin (BSA) in PBS for 5 minutes and then with anti-indole-3-acetic acid (IAA) mouse monoclonal antibodies (Sigma-Aldrich, St. Louis, MO, USA) diluted 1/100 in 0.1% BSA for 1 h at room temperature. After washing in PBS three times, 5 min each wash, the signal was revealed with ALEXA 488 (green fluorescence) conjugated anti-mouse antibodies (Molecular Probes, Eugene, Oregon, USA), diluted 1:25 in PBS for 45 min in the dark. After washing in PBS, the sections were counterstained with DAPI, mounted in Mowiol and observed in a confocal microscope. Confocal optical sections were collected using Leica TCS SP2 confocal scanning. Controls were performed by replacing the first antibody with PBS.

### Embryo conversion to plantlets: embryo germination

The procedure described by Prem et al. [29] was followed for *in-vitro* embryo conversion to plantlets (germination). Only microspore embryos originated from donor plants of low temperature growth chambers were used. MS media [30] containing 2% sucrose (w/v) gelled with 7 g/L bacteriological agar (w/v) was used for embryo germination. The effect of embryo induction temperature, desiccation treatment and incubation temperature for germination was tested in a four replicate 2 × 2 × 3 factorial CRD (embryos resulting from two different inductive temperatures × two desiccation treatments –slow air drying on sterile filter paper before incubation on germination media or direct incubation on germination media without air drying × three incubation temperatures for germination- 4°C for 10d in dark or 18°C in dark till activation of radicle and plumule or 25°C, 16 h photoperiod). The response was quantified after 15-20d of incubation in terms of percentage of embryos showing normal growth similar to zygotic embryo germination. The percentage data was arcsine transformed and analysed by 2 × 2 × 3 factorial ANOVA [27].

### Ploidy assessment by flow cytometry

The 3–4 leaf growth stage plantlets developed 25-30d after incubation on induction media were used for ploidy analysis using high throughput flow cytometry. Sample preparation buffer proposed by [31] was used for isolation of nuclei from young leaf samples. Leaf segments (7-10 mm diameter) were finely chopped using a sterile surgical blade in 1000 μL sample preparation buffer. The sample was then filtered using a 25 mm Swinnex® filter holder (Millipore) fitted with a double layer of 48 μM nylon filter and the final volume of the filtrate was adjusted to 700 μL with buffer. Thereafter, 70 μl Propidium Iodide (PI- 1.0 mg/ ml prepared in PBS) was added to the filtrate immediately followed by 0.75 μL RNase. The samples were incubated at room temperature for 10 min in dark and then analysed by a Becman Coulter FC500 flow cytometer equipped with an argon ion laser tuned at excitation λ = 488 nm and specific fluorescence signals were collected by 620 bp filter. The percentage data for ploidy level analysis for plantlets originating from microspore embryos induced at different temperature treatments was compared by two tailed *t*-test assuming unequal variance [32].

### Hardening and transplanting for haploid and doubled-haploid (DH) plant production

Following flow cytometric analysis, plantlets were hardened and transferred to pots in greenhouse (same conditions as for donor plants) following the procedure described by Prem et al. [29]. Observations were recorded for percentage of survival during the hardening and final realization of pod/ seed bearing plants to determine the overall efficiency of the DH protocol. The mean survival frequency of plants with different ploidy level was compared by two tailed *t*-test assuming unequal variance [32]. Throughout the manuscript data has been presented as means ± Standard Error (mean) and means represented by the same letter are not significantly different according to the stated criterion of comparison.

## Results

### Effect of growth temperature of donor plant and *in vitro* temperature treatments on triggering microspore embryogenesis

Microspore developmental stage and their structural organization at cellular level was analyzed (Additional file 1) in flower buds of different sizes. Buds containing the maximum proportion of vacuolated microspores, the most responsive stage for embryogenesis induction, were identified by DAPI-stained squash preparations of microspores (Additional file 1) and selected for *in vitro* purposes.

**Figure 1:**
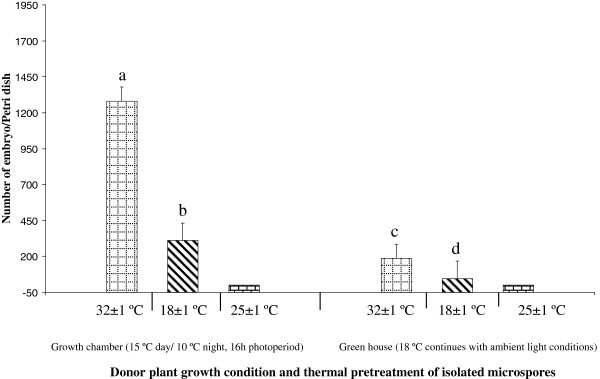
**Quantification of embryo formation in microspore cultures from two donor plants grown conditions and three temperature treatments*in vitro.*** The first three columns correspond to donor plants of growth chambers under 15°C day/10°C night, 16 h photoperiod. The last three columns correspond to donor plants from greenhouse grown under 18°C with ambient light conditions. Each histogram column represents the average number of microspore derived embryos obtained from three replicates wherein each replicate was defined by a minimum of two isolations and cultures. The total number of Petri dishes (n) observed were: 124 (1^st^ column), 119 (2^nd^ column), 123 (3^rd^ column), 132 (4^th^ column), 135 (5^th^ column) and 121 (6^th^ column). Histogram columns represented by different letters are significantly different from each other as per LSD_α=0.05_ and bars indicate SEm. Absence of error bars for 25 ± 1°C indicates no response

**Figure 2:**
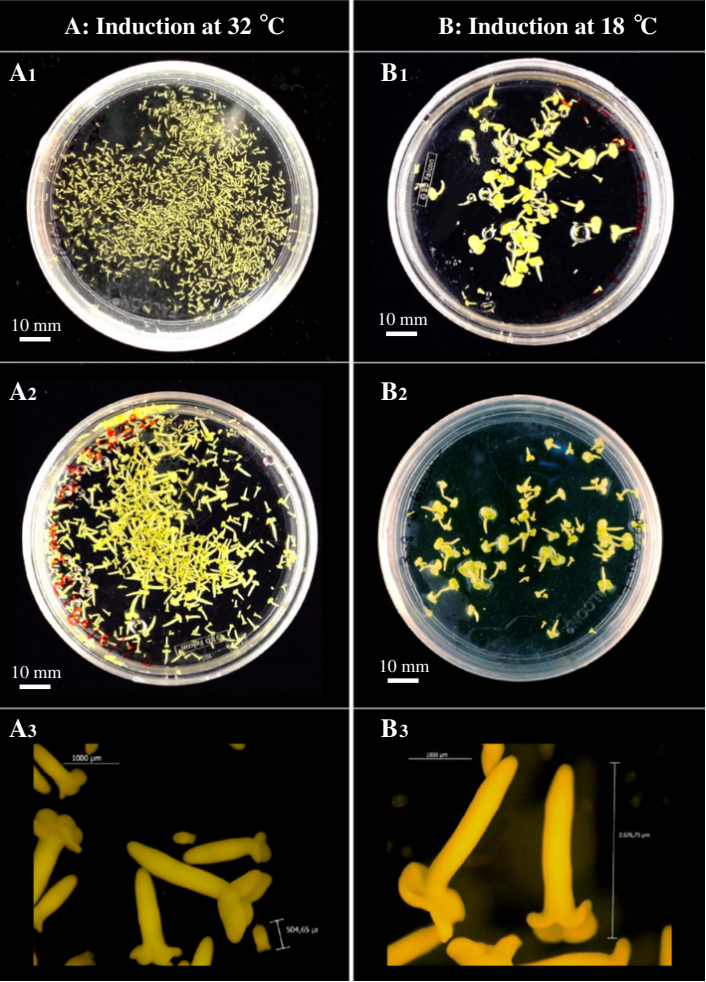
**Microspore embryo production from two donor plants growth conditions and two inductive temperature treatments*in vitro.*A**) Embryos induced by 32 ± 1°C treatment, culture at 30 days : (A1) embryos obtained from donors grown in growth chamber (15°C day/10°C night, 16 h photoperiod), and (A2) embryos obtained from donor plants grown in greenhouse (18°C with ambient light conditions). **B**) Embryos induced under 18 ± 1°C treatment, culture at 40 days: (B1) embryos obtained from donors grown in growth chamber, and (B2) embryos obtained from donor plants grown in greenhouse. (A3) and (B3): High magnification pictures showing the similar anatomy and different size of mature microspore embryos produced at 32 ± 1°C (A3, smaller embryos) and 18 ± 1°C (B3, larger embryos).

The effect of plant growth conditions and culture temperature treatment was studied in a two-fold experiment wherein microspores were collected from donor plants grown under two different environmental conditions (greenhouse at 18°C and growth chamber at 15°C day/10°C night) and isolated microspores were subjected to three temperature treatments 32°C, 25°C and 18°C. The cultures maintained at 25 ± 1°C did not result in any embryo induction (observations up to 40d), therefore this temperature was not considered for statistics. However, microspore embryogenesis was observed from Petri dishes cultured at 32.0 ± 1°C and 18 ± 1°C. Statistical analysis with factorial ANOVA was done for the 2 × 2 factors (two donor growth conditions and two *in vitro* temperature treatments). Both these factors had a significant effect on microspore embryogenesis, quantified in terms of number of embryos produced per Petri dish, as demonstrated by the ANOVA test which showed that the contribution of the two factors were statistically significant (Additional file 2). The embryo induction (Figure 1) was on an average, four times higher from microspores subjected to 32.0 ± 1 C (733.1 ± 149.6^a^ embryos/ Petri dish) compared to 18.0 ± 1°C (178.2 ± 68.0^b^ embryos/ Petri dish), irrespective of the donor plant growth condition (Figure 1). On the other hand, microspores obtained from plants grown under completely controlled environment in the growth chamber at low temperatures (15°C day/10°C night) showed seven times higher embryogenic ability (795.8 ± 146.4^a^ embryos/ Petri dish) compared to those obtained from greenhouse (115.4 ± 20.7^b^ embryos/ Petri dish) irrespective of the *in vitro* temperature treatment (Figure 1), therefore, in further analysis, only microspore cultures from donor plants of low temperature growth chambers were used. The highest embryogenesis response was recorded from microspores obtained from growth chamber donor plants and subjected *in vitro* to 32.0 ± 1°C (1281.1 ± 98.9 embryos/ Petri dish). Figure 2A and B show the comparison of embryo induction at different *in vitro* temperatures from donor plants grown under different conditions. At both *in vitro* temperatures, the mature microspore embryos showed similar morphology, characterised by clear differentiation of the cotyledons and radicle end, as shown in Figure 2A (32°C) and 2B (18°C). However, the size of the mature microspore embryos was smaller in Petri dishes subjected to 32°C (Figure 2A) compared to those induced at 18°C (Figure 2B), probably because the higher embryo density in the Petri dish at 32°C resulted in a higher competition for nutrition.

### Monitoring cellular organization during microspore embryogenesis induction and embryo development

#### Early stages of microspore embryogenesis induced at 18 ± 1°C

**Figure 3:**
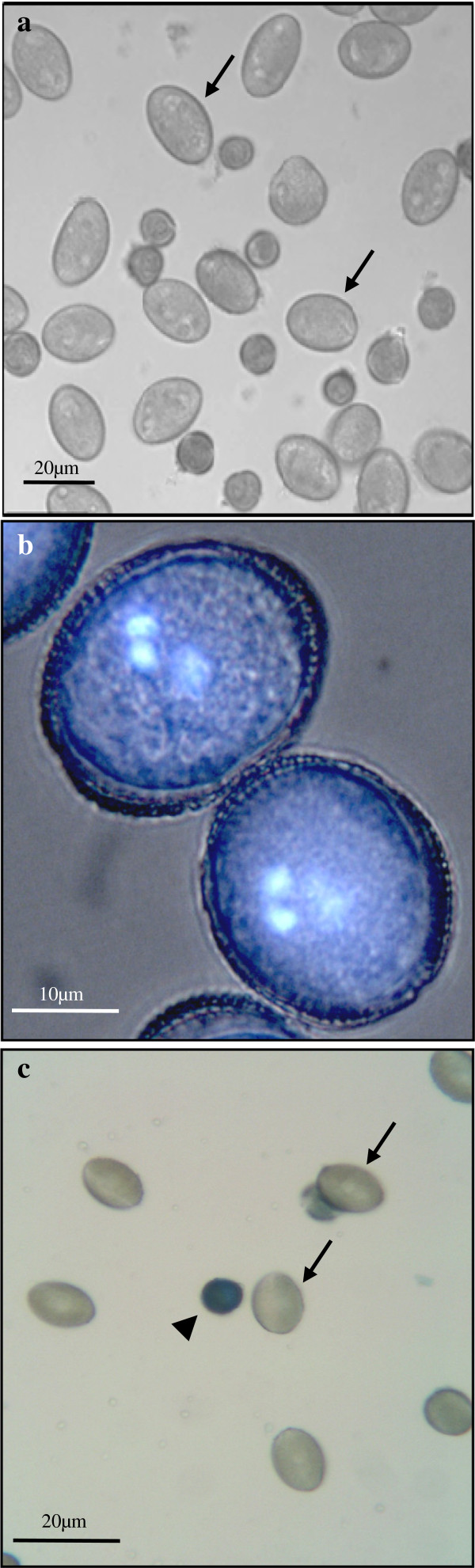
**Gametophytic- like developmental pathway in microspore cultures at 18°C.** Mature pollen-like structures (arrows) observed after 6-8d. **a**) Phase contrast micrograph at low magnification, **b**) High magnification micrograph of mature pollen-like cells observed under merge of phase contrast and DAPI epiflorescence, showing the two small sperm-like nuclei and the larger vegetative-like nucleus. **c**) Evan’s blue staining for detection of cell death showing the mature pollen-like structures unstained and the smaller microspores stained (arrowhead).

During the initial 6–8 days of culture at 18°C, the cell population in culture was predominantly composed of large oval cells (Figure 3a) which displayed three nuclei similar to mature pollen, as revealed by DAPI staining, two small nuclei similar in size and fluorescent brightness (sperm like nuclei) and one large nucleus (vegetative like nucleus) with faint fluorescence (Figure 3b). Also, numerous small rounded microspores were observed in the culture (Figure 3a). Many of them were stained when treated with Evan’s blue, specific staining for dead cells (Figure 3c), indicating the existence of a high proportion of dead cells in the culture.

**Figure 4:**
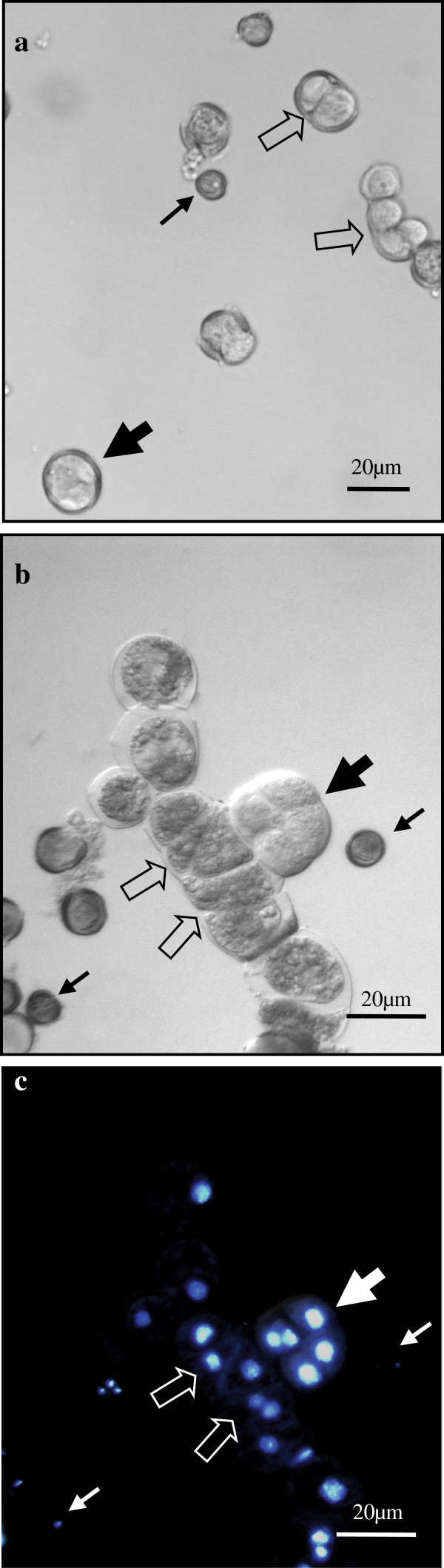
**Different structures and embryogenesis pathways in microspore cultures at 18°C.** Panoramic views of cultures at 13 days (**a**) and 17 days (**b**, **c**) showing different structures and embryogenesis pathways at the same time points: small dead cells (thin arrows), rounded multicellular structures surrounded by exine (thick arrows) and uniseriate structures (open arrows) of two-three cells at 13 days (**a**) and longer structures at 17 days (b, c). **b**, **c**) Phase contrast (**b**) and DAPI epifluorescence (**c**) views of the same microscopic field.

**Figure 5:**
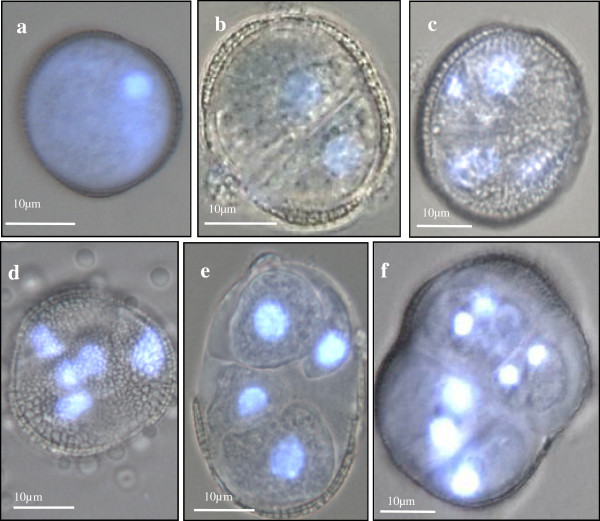
**Early stages of the microspore embryogenesis pathway without suspensor induced at 18°C.** Micrographs of phase contrast and DAPI epiflorescence to visualize the nuclei. **a**) Vacuolated microspore immediately after isolation, **b**) Two cell pro-embryo after 13-15d, **c**-**f**) Multicellular pro-embryos surrounded by the exine which appeared intact or just broken, displaying increasing number of cells (4–10 cells), and observed after 17-19d.

**Figure 6:**
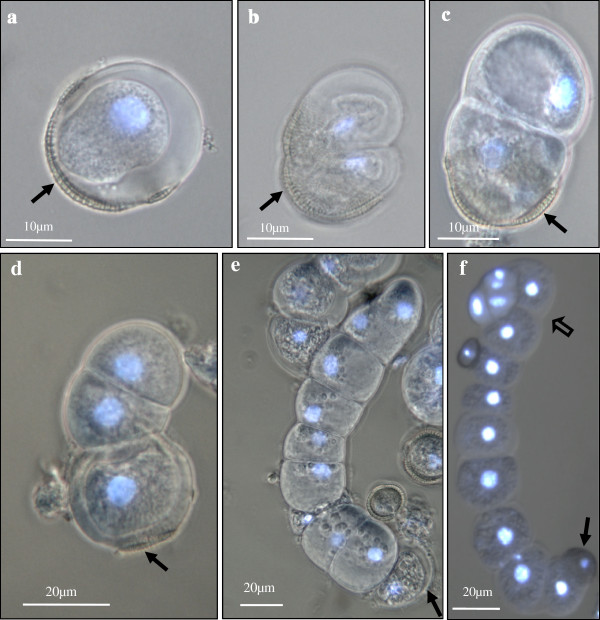
**Early stages of the microspore embryogenesis pathway with suspensor-like development induced at 18°C.a**-**d**) Cultures at 13–15 days, **e**-**f**) Cultures at 17–19 days, Phase contrast micrographs with DAPI epifluorescence to visualize the nuclei. **a**) Enlarged single cell with broken exine (arrow). **b**-**c**) Two cell pro-embryos with the exine remnants (arrows) attached to one of the cells. **d**-**e**) Three-cell and seven-cell suspensor-like structures with the exine attached to the original microspore located at one pole of the structure, indicated by arrows. **f**) Pro-embryo with a four-six cell globular head (open arrow) and six-eight cell suspensor-like structure with the exine attached to the terminal end (arrow).

Within the next 13–19 days, the culture was composed of different structures (Figure 4) that could be grouped in three types of developmental programs, together with many small dead microspores (Figure 4, thin arrows). These structures were, a) gametophytic-like cells (Figure 3) that resembled the mature pollen, as described above, b) rounded two-cell and multicellular pro-embryos, many of them surrounded by the microspore wall, the exine (thick arrows in Figures 4 and 5), similar to those of the microspore embryogenesis system induced by continuous 32°C, and c) structures with an uniseriate organized pathway of divisions leading to the development of suspensor-like structures henceforth referred to the suspensor-like pathway (open arrows in Figures 4 and 6).

The cell population of the suspensor-like pathway was highly asynchronous during the 13-19d period, consisting of three main types of structures: large single cells with large nucleus, a very thick cell wall surrounded the cell, and ruptured exine (Figure 6a), two-cell pro-embryos with broken exine (Figure 6b, c), and 3–8 cell suspensor-like structures with the exine attached to the original microspore (Figure 6d, e), some of them displaying a distinct proembryo globular head of 4–6 cells at the opposite pole (Figure 6f). The smaller structures of 1–3 cells were mainly observed between the 13-15d,, and larger pro-embryos with suspensor-like structures were predominantly observed between 17-19d.

#### Differential cellular features between suspensor-like and embryo structures

**Figure 7:**
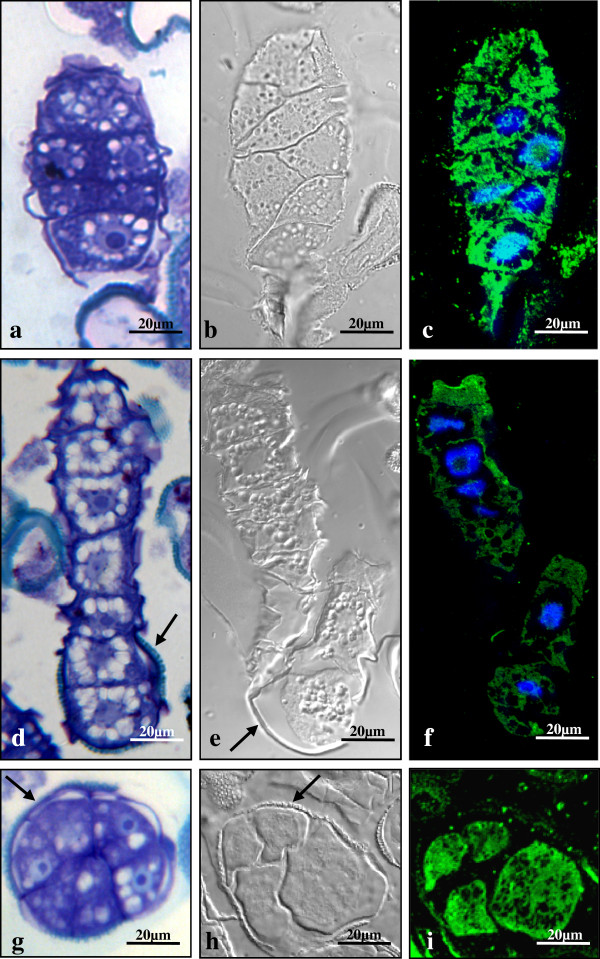
**Differential features of embryo and suspensor-like cells from two developmental pathways induced at 18°C.a**-**f**) Suspensor-like pathway: individual micrographs showing the embryo head (**a**-**c**) and the suspensor-like structure (**d**-**f**). **g**-**i**) Developmental pathway without suspensor: rounded multicellular embryos surrounded by the exine. **a**, **d**, **g**) Subcellular organization observed in semithin sections stained with toluidine blue. DIC images (**b**, **e**, **h**) and anti-auxin immunofluorescence (**c**, **f**, **i**) of the same structures. Exine remnants are pointed by arrows. Embryo cells of both pathways showed similar organization with dense cytoplasms (**a, g**), and high IAA immunofluorescence signal (**b, c, h, i**), while suspensor-like cells displayed numerous vacuoles surrounding the central nucleus (**d**), and low IAA signal (**e, f.**)

The microscopic analysis of semithin sections of resin-embedded samples revealed the subcellular organization of the embryo and suspensor-like structures (Figure 7a, d), The suspensor-like structure (Figure 7d) was formed by a uniseriate line of cells separated by straight cell walls and containing large central nuclei surrounded by numerous vacuoles separated by thin radial cytoplasmic threads, typical organization of the suspensor cells in early zygotic embryogenesis [33]. Remnants of the exine were observed surrounding the cell localized at one pole of the suspensor structure (Figure 7d). The cells forming the embryo structure at early stages of development displayed polygonal shape and dense cytoplasms (Figure 7a). Analogous cellular organization was found in embryos of the other pathway, observed as rounded multicellular structures surrounded by the exine and without suspensor (Figure 7g).

In order to analyze characteristic differential features of embryo and suspensor cells in the structures developed in the cultures, intracellular localization of endogenous auxin was studied by immunofluorescence with specific anti-IAA antibodies. Confocal microscopy analysis showed a low IAA signal in the suspensor-like cells (Figure 7e, f) while an intense IAA fluorescence was observed in the embryo cells (Figure 7b, c), indicating a differential localization of endogenous auxins between suspensor-like and embryo cells at this early developmental stage, being the auxin concentrated in the embryo, as occurs in zygotic embryogenesis. Multicellular embryos surrounded by exine also showed high and homogeneous IAA localization (Figure 7h, i),

#### Quantitative analysis of developing structures at early stages in microspore cultures at 18°C

**Figure 8:**
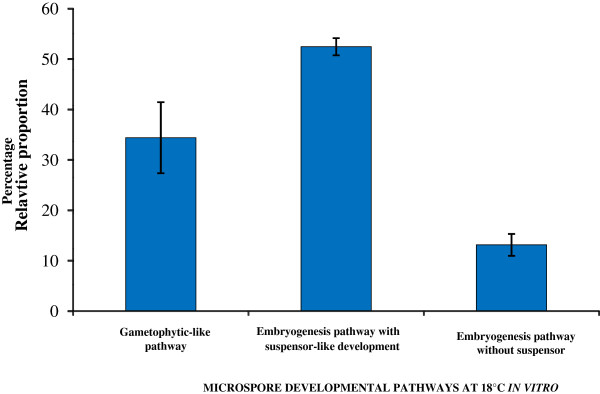
**Quantification of the occurrence of different developmental pathways in microspore cultures at 18°C, after 13–15 days.** Each histogram column represents the percentage of microspores that follow a particular developmental pathway: the gametophytic-like pathway, the embryogenesis pathway with suspensor-like development, and the embryogenesis pathway without suspensor. Bars indicate SEm.

**Figure 9:**
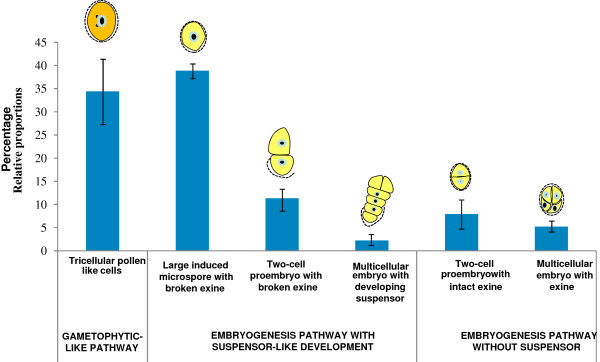
**Quantification of the presence of different structures from various developmental pathways occurring in microspore cultures at 18°C, after 13–15 days.** Each histogram column represents the percentage of particular structures indicated by schematic diagrams above each column, representing different developmental pathways: Gametophytic-like development and early stages of the two microspore embryogenesis pathways, with suspensor-like development and without suspensor. Bars indicate SEm.

In order to analyze the cell population dynamics in the three different developmental pathways observed, a quantitative analysis of the different structures present in the cultures was carried in cultures at 13-19d using DAPI stained preparations, eliminating the dead cells of the analysis. The results (Figure 8) showed a large predominance of the suspensor-like pathway (52.4%) over the other two pathways, the gametophytic-like pathway (34.4%), and the embryogenesis pathway without suspensor, similar to that usually found under 32°C (13.1%). The percentage of the different structures of each developmental pathway was also evaluated and is schematically represented in Figure 9.

#### Late microspore embryos development at 18 ± 1°C

**Figure 10:**
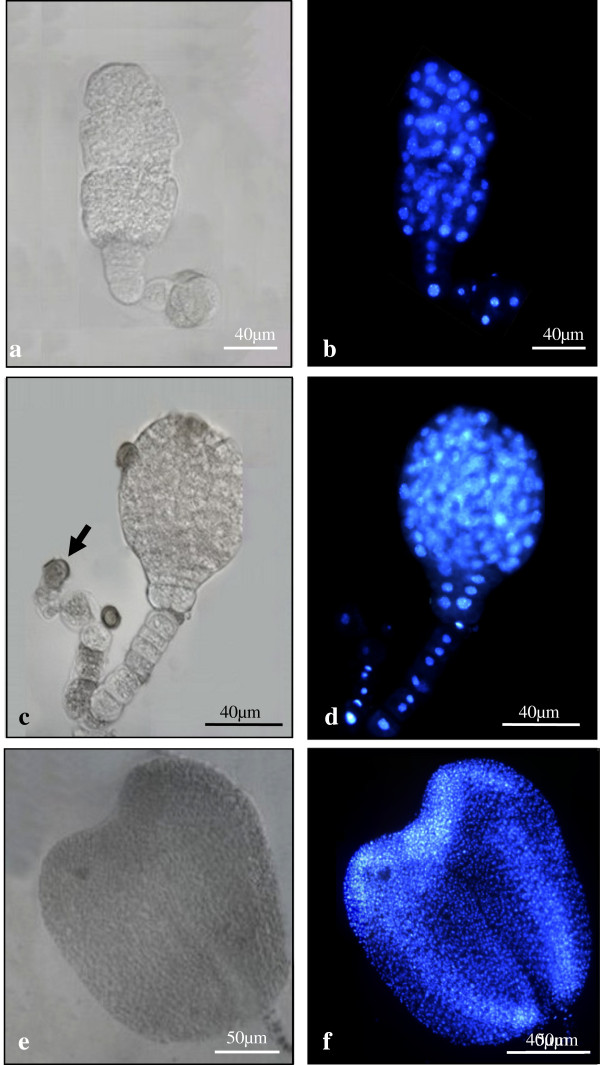
**Late stages of the microspore embryogenesis induced at 18°C: globular, and heart-torpedo embryos.a**-**d**) Globular embryos with long suspensor like structures (26-30d) **e**, **f**) Heart-torpedo shaped embryo with suspensor-like structures (28-30d). Micrographs from squash preparations, the same embryo was observed under phase contrast (**a, c, e**), and DAPI epiflorescence for visualization of the nuclei (**b, d, f**).

At 20-24d, two types of multicellular embryos were observed: small globular pro-embryos without suspensor and proembryos formed by a distinct globular head of 6–8 cells and an uniseriate suspensor-like structure with 8–10 cells. During the next 26-30d, embryos displayed a much larger globular head composed of cells which had divided in various directions (Figure 10). In most of these embryos with suspensor, the remnants of the exine were found attached to the filamentous end opposite to the globular head (Figure 10c). The growth and elongation of the suspensor was sustained throughout the late globular to heart shaped stage (Figure 10). Further development produced torpedo embryos which did not show suspensor cells. The mature microspore embryos induced at 18°C were similar to those observed at 32°C, although these developed much later, after 34-40d of culture.

### Germination of microspore embryos induced at both temperatures 32°C and 18°C

**Figure 11:**
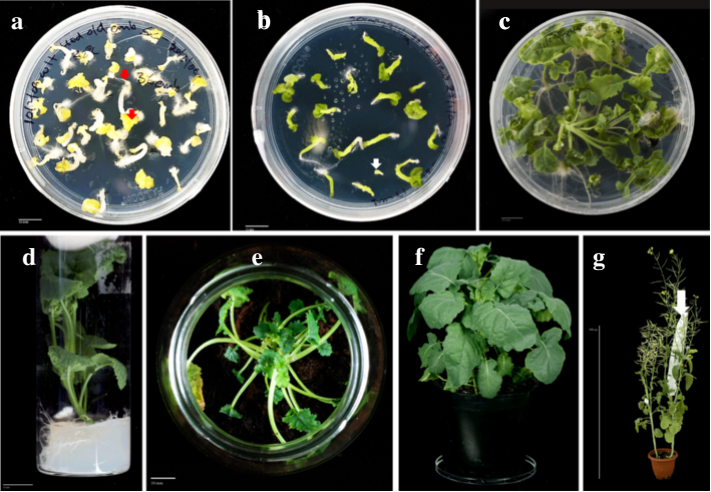
**Conversion of microspore derived embryos to plants.a**, **b**, **c**) Consecutive stages during embryo germination; **a**) Slow air-dried embryos cultured at 18 ± 1°C which show distinct bipolar zygotic embryo-like germination in terms of simultaneous development of radicle and plumule end (indicated by red arrows) within 20d of incubation, b) **b**) Activation of chlorophyll and green pigmentation of cotyledons/embryo axis as a response to exposure to light (10-12d) after 20d dark incubation at 18±1°C. White arrow indicates a non-responsive/dead embryo, **c**) 2–3 leaf growth stage plantlets obtained after 21-28d culture at 25 ± 1°C, 16 h photoperiod. **d**, **e**, **f**, **g**) Consecutive stages during plantlet growth and transfer to photoautotrophic conditions; **d**) Plantlet elongation and development on MS medium, **e**) Transfer of plantlets to agropeat in glass jars for hardening, **f**) Regenerated plant transferred to greenhouse originated from plantlets at the vegetative growth stage (6–9 leafs), **g**) Flowering and further growth of a mature spontaneous DH plant. Arrow indicates bags placed for self-pollination.

The embryos induced by the two temperatures (32°C and 18°C), from donor plants grown under low temperature conditions in growth chambers, were tested for their germination ability, under different desiccation treatments (slow air desiccation or direct culture without desiccation) and different temperature conditions (4°C for 10d, 18°C up to activation of radicle–plumule, and 25°C continuously). The results of this experiment were quantified in terms of percentage of direct germination (Table 1) and a 2 × 2 × 3 ANOVA test was applied to elucidate interaction among factors (Additional file 3). While no statically significant difference was observed between the germination response of embryos induced under 32°C and 18°C treatments, the desiccation treatment and the temperature for germination had a significant influence on germination (Additional file 3). The quantitative analysis of embryo germination (Table 1) showed that slow air desiccation resulted in more than three times higher (50.3 ± 19.8%) embryo germination percentage compared to direct culture of embryos (14.0 ± 4.7%), irrespective of the embryo induction temperature and conditions for incubation. The optimum frequency of embryo germination (86.5 ± 3.7%) was obtained by slow air desiccation of embryos followed by their culture at 18.0 ± 1°C in dark conditions till activation of radicle and plumule (Table 1). Under these conditions, the plumule (cotyledons) end of embryos turned yellow and visible development of radicle was observed (Figure 11a), within 20-24d of culture in germination media. These embryos were then shifted to 25.0 ± 1°C, 16 h photoperiod conditions where chlorophyll activation occurred within the following 7-10d of culture (Figure 11b). Three-four true leaf stage plantlets were obtained after about 24-28d (Figure 11c) and leaf disk segments from these plantlets were used for flow cytometry to analyse ploidy level and haploid/spontaneous doubled haploid plantlets were labelled before further growth.

**Table 1:** Interaction between temperature and desiccation treatments for microspore embryo germination

**Germination temperature treatment**	**Percentage of germination at different desiccation treatments**	
	**Air drying (307)**^**#**^	**Direct culture (298)**
4°C- 10 d, dark (207) ^#^	*46.3 ± 3.3^b^	7.0 ± 1.1^c^
18°C, dark till activation of radicle and plumule (204)	86.5 ± 3.7^a^	23.0 ± 2.1^a^
25°C, 16 h photoperiod (194)	18.1 ± 2.5^c^	12.1 ± 1.5^b^
Mean (irrespective of germination temperature treatment)	50.3 ± 19.8	14.0 ± 4.7

Data from embryos obtained from microspores of donor plants grown under low temperature conditions in growth chambers.

*Percentages are presented for four replicates for each factor (Percentage: mean ± SE_m_).

^$^Means represented by the same small case letter in each column are not significantly different as per LSD _(α=0.05)_.

^#^Values in parenthesis represent the total number of embryos observed (*n*).

### Conversion of microspore-derived plantlets to mature seed bearing plants

The three-four leaf stage plantlets were further sub-cultured in tubes and further leaf development and shoot elongation was observed in the following 20-21d of sub-culture (Figure 11d). At this stage, plantlets were subsequently transferred to glass jars containing agropeat with half-strength MS media without sucrose for acclimatisation/hardening. Under these conditions, within the next 21-30d, shoot elongation, multiplication of leaves (15–20 leaves) and visible root growth was observed. Such photoautotrophic plantlets were subsequently transferred to plastic pots (Figure 11e) and grown in a growth chamber as described for donor plants. Subsequently, the plants were shifted to greenhouse after crown initiation (Figure 11f). A total of 86 plants were transferred to greenhouse and high survival frequency ranging from 96.4 to 98.0% was recorded, irrespective of their ploidy status, up to the flowering stage (Figure 11g).

### Ploidy level of plantlets regenerated from microspore derived embryos

**Figure 12:**
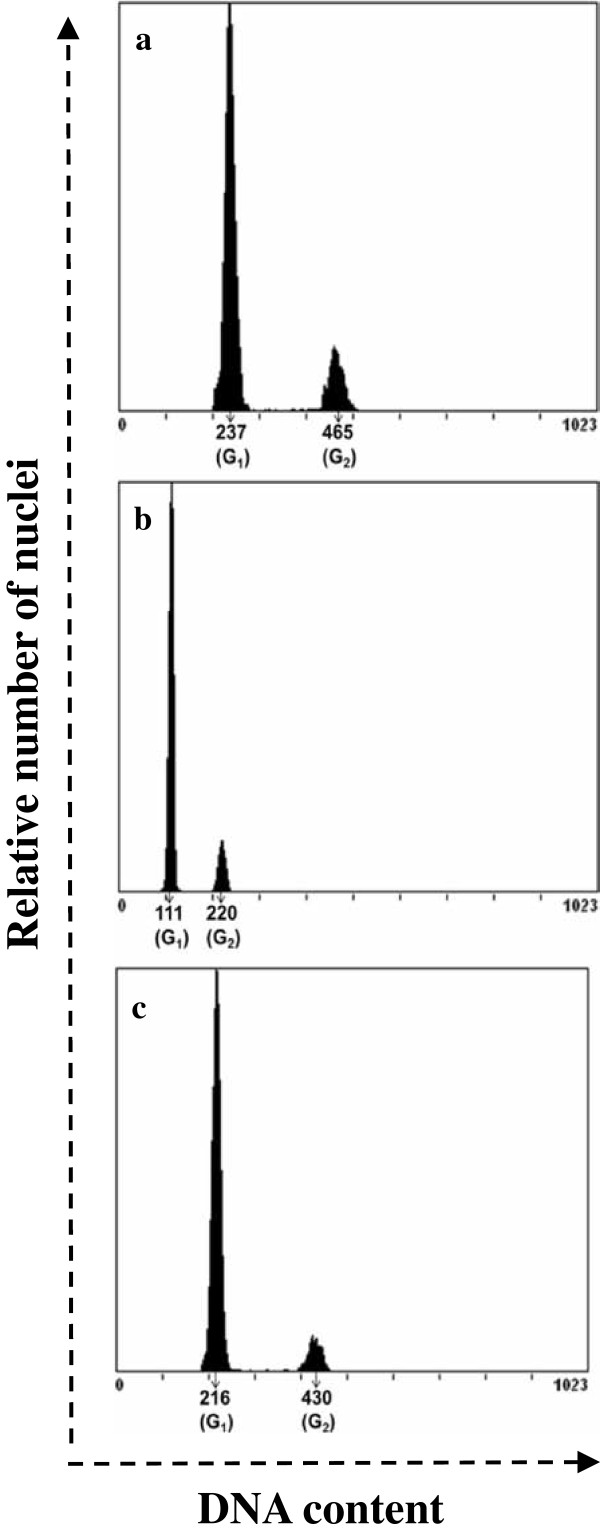
**Flow cytometry analysis of the ploidy level of representative diploid (control), haploid and spontaneous doubled haploid plants regenerated from microspore embryogenesis cultures.** Ploidy levels were determined from propidium iodide stained-nuclei isolated from leaf discs of 4–5 leaf growth stage plants. Flow cytograms generated from a diploid donor plant (**a**), a haploid (**b**), and a spontaneous doubled haploid (**c**) plant regenerated from microspore cultures respectively. In the three histograms, mean channel values are presented for peaks corresponding to G1 and G2 phases of cell cycle. In the diploid control (a) and the spontaneous doubled haploid (c), G1 and G2 peaks appear at similar DNA content channels, whereas in the haploid sample (b), the relative DNA content is the half, the G1 and G2 peaks appear to almost half mean channel values than those of the diploid control.

The plantlets regenerated from microspore embryos obtained from the two different temperature conditions were analysed for ploidy level by flow cytometry, standarizing a novel method of nuclei isolation which resulted in high fidelity separation of nuclei. Flow charts generated from diploid controls (from a donor plant), haploid and spontaneous doubled haploid plants from microspore embryos were obtained (Figure 12) and quantified A percentage of 63.9 ± 9.9% of the regenerated plants were spontaneous doubled haploid irrespective of the induction temperature used (Table 2). Embryos obtained at 18°C produced a higher percentage of haploid plants (41.3 ± 6.5%) than the embryos induced at 32°C (33.3 ± 13.3%). No statistical difference was observed for recovery of spontaneous doubled haploids from microspore embryos derived from 18.0 ± 1°C and 32.0 ± 1°C (Table 2). The quantification of the efficiency of hardening and plant growth till flowering of haploid and doubled-haploid plants is shown in Table 3. After transfer to greenhouse and further growth, most regenerated plants reached the flowering stage and, among them, the spontaneous diploids had similar hardening survival (96.1 ± 3.8%) compared to the haploid plants (91.6 ± 1.6%).

**Table 2:** Quantification of haploids and doubled-haploids plantlets regenerated from microspore embryos obtained from two different temperature treatments

**Temperature for embryogenesis induction**	**Plantlets tested**	**Number of plantlets**	
		**(**^**#**^**Percentage: mean ± SE**_**m**_**)**	
	**(*n*)**	**Spontaneous DH**	**Haploids**
18°C	46	27	19
		(58.6 ± 6.5)^a^	(41.3 ± 6.5)^a^
32°C	30	20	10
		(66.6 ± 13.3) ^a^	(33.3 ± 13.3)^b^
Independent of embryogenesis induction temperature	96	63.9 ± 2.6	37.3 ± 4.0

^#^Percentage is presented for two replicates of flow cytometry analysis consisting of 15–23 plants each (total = n). In order to check for experimental bias, plantlets were selected at random from regenerated embryos produced from different microspore isolation.

Means of percentages represented by the same small case letter within each column are not significantly different as per two tailed *t*-test assuming unequal variance at α = 0.05 for spontaneous DH or Haploid percentage.

**Table 3:** Quantification of the efficiency of hardening and plant growth till flowering of haploid and doubled-haploid plants regenerated from microspore culture

**Ploidy of plantlets**	**Hardening**		**Plant growth till flowering**	
	**Total number of plants**^**#**^	**Percentage of survival**^**$**^	**Total number of plants**^**#**^	**Percentage of survival**^**$**^
Spontaneous DH	51	96.1 ± 3.8^a^	49	98.0 ± 2.0^a^
Haploid	35	91.6 ± 1.6^b^	33	96.4 ± 3.5^a^

^#^9-26 plants tested in two replicates.

^$^ Percentage: mean ± SE_m_.

Means represented by the same small case letter are not significantly different as per two tailed *t*-test assuming unequal variance at α = 0.05 for survival during hardening or plantlet growth till flowering.

## Discussion

### Donor plant growth at low temperatures significantly improves yield of microspore embryogenesis

Several studies have shown the influence of donor growth environment on microspore totipotency [34-38]. The reasons why the microspores in the same stage of development obtained from different growth environment react in different ways to the same stimulus still remain largely unknown. Donor plant growth in controlled environment specially subjected to low temperature, as in the present study, have been reported to have a slower metabolic rate compared to partially controlled/ field grown donors, due to altered endogenous levels of growth regulators and nutrient utilization of anthers and microspores [39].

Our results showed a better embryogenic response of microspores from donor plants grown at low temperatures, Under low temperature the *in vivo* development of microspores would be slower and therefore, the general asynchrony of microspores inside anthers would be lower, which would favour to collect a more homogeneous population of late vacuolated microspores, exactly in the small developmental window of maximum embryogenic response. On the other hand, during pollen development *in vivo*, any nutritional reduction or environmental change can affect pollen development and viability. Our results showed that donor plants subjected to strictly controlled low temperature conditions in the growth chambers would contain a higher proportion of microspores at optimum metabolic state and therefore exhibit a higher embryogenic response than those from greenhouse donors.

### 18°C stress treatment efficiently induces microspore embryogenesis through different developmental pathways

**Figure 13:**
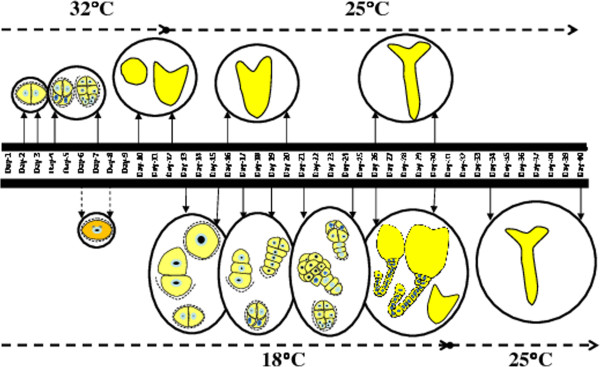
**Schematic representation of the timing of the microspore embryogenesis developmental pathways in the two*in vitro*systems: under 32°C and 18°C treatments.**

In *B. napus*, isolated microspore development at 18°C has been studied for *in vitro* gametophytic development [9,10], however, these reports only illustrated cellular events from the first week up to 10d of culture, until the development of mature pollen-like structures, as observed in the present work. The detailed analysis of long term low temperature incubation of isolated microspores has not been studied as yet. The present results reveal a novel mechanism for microspore embryogenesis induction in *B. napus* using continuous low temperature stress treatments. Based on our results, the Figure 13 shows the schematic summary of the timing of the different developmental pathways of microspore embryogenesis in the two different temperature treatments, the one at 32°C and the new system at 18°C. The major embryogenesis pathway found under 18°C treatment involves the early polarity establishment and the formation of suspensor-like structures.

The fact that temperature can control the development of isolated microspores in culture has been well documented in *B. napus*, Lower temperatures favour asymmetric divisions while heat treatments switch symmetric divisions [6,9,10]. In the new system developed here, during the first 6–8 days low temperature would promote gametophytic development and an asymmetric division forming vegetative and generative-like cells, whereas at longer periods low temperature favours an embryogenesis pathway whose first division originates asymmetric cell identities.

The new *in vitro* system, under low temperature, efficiently induced microspore embryogenesis by two different developmental pathways, the major pathway involving the formation of suspensor-like structures, and the minor pathway producing embryos without suspensor, similar to the development usually found under 32°C. The embryogenesis efficiency of the new system at 18°C was high, but lower than that at 32°C. This quantitative difference between both systems could be due to the proportion of microspores following the gametophytic-like pathway, not present at 32°C, together with the cell death occurring in the first days. The variation observed in embryo size would be related to the number of embryos produced per Petri dish at both temperatures, 32°C and 18°C, and could be due to *in vitro* competition for nutrition. Similar morphological variation in microspore embryos has been reported for *B. napus*[40] and *B. juncea*[1].

One interesting feature found in the microspores following the suspensor-like pathway was the formation of a thick cell wall under the exine which was observed at very early stages, in the embryogenic microspores with large size and ruptured exine. Changes in the inner wall of the microspore have been reported after embryogenesis induction in different systems [16-18]. These special cell walls of embryogenic microspores usually showed an increased thickness with respect to the original microspore wall, therefore being suggested as embryogenic markers [16-18]. The observation of a thick inner wall in the large microspores with broken exine under 18°C, constitutes an additional evidence supporting the embryogenic nature of these microspores.

### A microspore embryogenesis pathway occurring at 18°C involves early polarity establishment and suspensor development, mimicking zygotic embryogenesis initials

During zygotic embryo development in *B. napus*, the polarity of developing embryo is established at the first cell division and the suspensor develops from very early stages showing 6–8 cells at the octant phase of the embryo proper [33,41,42]. Several reports have emphasized on the similarity of zygotic and microspore embryogenesis during later stages, e.g. from globular embryos and further development [16,17,33,42,43]. In the new system reported here, the most striking similarity between the zygotic and microspore embryogenesis was the occurrence of suspensor-like cells at very early stages. The occurrence of suspensor-like cells has been reported as sporadic and unpredictable after a 32°C treatment, only at later stages, in microspore globular and heart embryos. [33,42]. It had been proposed that the suspensor-like cells which appear in globular-microspore embryos induced at 32°C develop from hypophysal-like cells of the globular embryo [33,42,44]. More recently, Joosen et al. [11] have reported the development of a novel method of microspore cultures, induced by a 24 h, 32°C heat shock which lead to embryo development that closely resembles zygotic embryogenesis. Further, Supena et al. [12] have reported the detail of this zygotic-embryo-like pathway. Our results revealed the occurrence of a predominant suspensor-like pathway under a different temperature treatment, 18°C; in this pathway, the first divisions of the microspore would originate an elongated structure from which the distal part generates a globular embryo proper while the lower part resembles a suspensor, establishing an early polarity in the microspore embryos development and mimicking zygotic embryogenesis at very early stages. Our results revealed that the cells forming these two parts of the microspore-derived structures displayed different cellular organization and IAA concentration, similar to suspensor and embryo cells [33,45], therefore confirming their different cell type and fate. Endogenous auxin concentration was also found in multicellular embryos surrounded by exine and without suspensor, indicating that auxin is present in microspore-derived embryo cells, independently of the pathway followed. In the zygotic embryogenesis, following the initial asymmetric zygote division, the identities and cell division patterns of the basal and apical cells, which will form the suspensor and embryo proper respectively, are determined by differential expression of some transcription factors and proteins (reviewed in [19]). In the new suspensor-like microspore embryogenesis pathway, the first division formed two equally sized daughter cells which could led to asymmetric cell identities, as in other plant processes in which different cell fates are determined in equally sized daughter cells by polar localization of inner and external signals [19,20]. The detection of a higher concentration of endogenous IAA in the embryo cells in comparison with the suspensor, as reported here, suggests that an early auxin gradient could be one of the inner signals which would contribute to the identity specification of suspensor and embryo cells in this microspore embryogenesis system, like in zygotic embryogenesis [45,46].

The mechanisms breaking the symmetry of cell division [20] would not act in the reprogrammed microspores which did not develop suspensors. In zygotic embryogenesis, the absence of specific signals, like MAPKKK-YODA (YDA) or Short Suspensor protein (SSP), in *yda* and *ssp* mutants makes zygotes to fail in expand longitudinally, the first zygotic division is symmetric and further divisions occur in random orientation, therefore the suspensor does not develop and embryo develops normally until octant stage [47]. Together with extrinsic factors, there are also evidences for intrinsic factors, mainly transcriptional regulators, which determine differential expression of factors of asymmetric gene expression in two-cell embryos (De-Smet and Beckman [19]).

The reason why microspores under the same external *in vitro* conditions showed such different behaviours remains unknown; differences in the initial developmental stage and metabolic state of the microspores could affect the reception of signals to follow different types of developmental programs and for establishment of asymmetric identities and suspensor cell fate specification in some microspores, like it occurs in zygotic embryogenesis [20]. At later stages of development, our report shows the morphological similarity of mature microspore embryos produced from the two inductive treatments (32°C and 18°C), the suspensor is not more found at later stages in any *in vitro* system, indicating its role at early stages. This new *in vitro* system represents an advantage for a comparative study of the mechanisms of both embryogenesis pathways, since it permits to analyze different cellular dynamics at the same conditions of induction, studies that would shed light on the molecular mechanisms underlying microspore reprogramming to embryogenesis and the establishment of polarity and developmental embryo patterning.

### High efficiency in microspore embryo germination and haploid/doubled-haploid plant regeneration achieved by a new *in vitro* system

In the present work, the development of a novel protocol for high frequency microspore embryo conversion to mature seed bearing plants has been achieved. Controlled air desiccation and low temperature shock has been reported for increasing embryo germination frequency in *B. napus*[40,48,49]. Cold shock of embryos has been reported to enhance germination frequency of microspore embryos up to 90% [49], however, the plantlet development frequency remains up to 50-58% [50,51]. Desiccation of microspore embryos with or without pre-treatment with abscisic acid (ABA; 50 μM) has been reported to enhance conversion frequency in *B. napus*[40,52]. Embryo drying has a significant role in regulating endogenous ABA levels that affect the maturation and conversion processes [53]. In our study, a combination of air desiccation followed by incubation of mature microspore embryos at 18°C in dark resulted in more than 90% germination frequency; this treatment for germination of microspore embryos has not been reported in *B. napus* doubled-haploid (DH) systems. Moreover, the overall efficiency of the hardening and transplanting achieved by us was higher than 95% for both haploid and spontaneous DH plants. On the other hand, our results also prove the physiological similarity between the microspore embryos produced by two different induction systems (32°C and 18°C). The reports cited above specifically comparing microspore embryogenesis and zygotic embryo development, only highlight the morphological similarities and did not detail physiological equivalence in terms of embryo germination capability, as our present work revealed.

## Conclusions

The present work reveals a novel mechanism for efficient microspore embryogenesis induction in *B. napus* using continuous low temperature treatment. Results indicated that low temperature applied for longer periods favours an embryogenesis pathway whose first division originates asymmetric cell identities, early polarity establishment and the formation of suspensor-like structures, mimicking zygotic embryogenesis. The new *in vitro* system reported here provides a convenient tool to analyze *in situ* the mechanisms underlying different developmental pathways during the microspore reprogramming, breaking or not the cellular symmetry, the establishment of polarity and the developmental embryo patterning, which further produce mature embryos and plants.

## Abbreviations

DH, Doubled haploids; CRD, Completely randomized design; IAA, Iindole-3-acetic acid; PI, Propidium iodide; MS media, Murashige and skoog medium; ABA, Abscisic acid; DIC, Differential interference contrast.

## Competing interests

The authors declare that they have no competing interests.

## Authors’ contributions

DP performed most of the experimental analyses, the statistics and quantification studies, and wrote the first manuscript draft. MTS had an active contribution to the *in vitro* assays and performed some microscopic analysis. IB and HR performed the auxin immunofluorescence essays. IB also helped in initial flow cytometry analysis, formatted and prepared several figures. MCR participated in the design and coordination of the work, helped to draft the manuscript and discussed the results. PST designed and coordinated the experimental work, designed and contributed to the draft and wrote most of the manuscript and its revision. All authors read and approved the final manuscript.

## Supplementary Material

Additional file 1 ***In vivo*microspore gametophytic development in*Brassica napus.*** Developmental stages from donor plants grown under low temperature conditions in growth chambers. Semi-thin sections stained with toluidine blue showing the structural organization of the young microspore (a), vacuolated microspore (b) and bicellular pollen (c). Squash preparations of anthers stained with DAPI and revealing the nuclei of others microspores and pollen at the same developmental stages, young microspore (a’), vacuolated microspore (b’) and bicellular pollen (c’). (PDF 32 kb) Click here for file

Additional file 2 **Statistical analysis of the contribution of several factors to embryo production in microspore cultures.** 2 × 2 factor ANOVA for partitioning variance for microspore embryogenesis response of buds collected from donor plants grown in two different conditions and after isolation, subjected to two different thermal stress conditions (8 replicate data). (PDF 67 kb) Click here for file

Additional file 3 **Statistical analysis of the contribution of several factors to microspore embryo germination.** 2 × 2 × 3 factorial ANOVA for partitioning variation in response of embryo conversion to plantlets (germination) for microspore embryos obtained from two different pathways and from donor plants of growth chambers, cultured directly or air-desiccated and subjected to three different germination pre-treatments. (4 replicate each with 7–20 embryos cultured per replicate for each factor). (PDF 39 kb) Click here for file
