# Supplementary figures and images for: A new microspore embryogenesis system under low temperature which mimics zygotic embryogenesis initials, expresses auxin and efficiently regenerates doubled-haploid plants in Brassica napus
Source: BMC Plant Biol. 2012 Aug 2;12:127. doi: 10.1186/1471-2229-12-127 (PMC3464609; doi:10.1186/1471-2229-12-127)

**Additional file 1**

***In vivo* microspore gametophytic development in *Brassica napus***

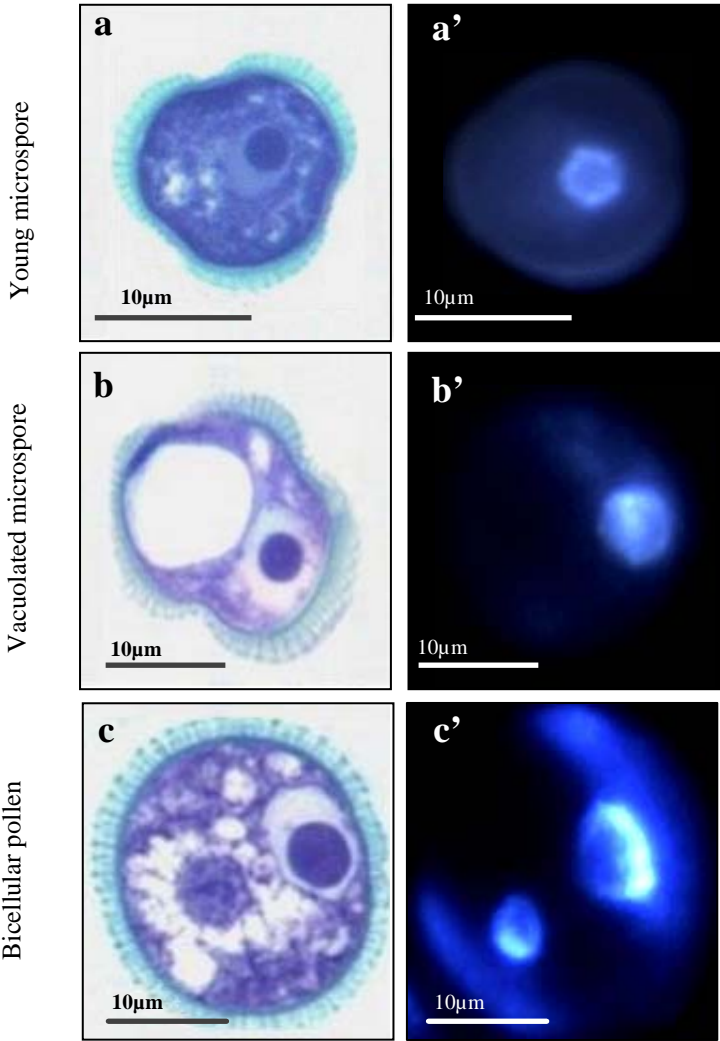

Supplement: Additional file 1 — In vivomicrospore gametophytic development inBrassica napus. Developmental stages from donor plants grown under low temperature conditions in growth chambers. Semi-thin sections stained with toluidine blue showing the structural organization of the young microspore (a), vacuolated microspore (b) and bicellular pollen (c). Squash preparations of anthers stained with DAPI and revealing the nuclei of others microspores and pollen at the same developmental stages, young microspore (a’), vacuolated microspore (b’) and bicellular pollen (c’). (PDF 32 kb) [file 1471-2229-12-127-S1.pdf]
