# Supplementary material for: A new microspore embryogenesis system under low temperature which mimics zygotic embryogenesis initials, expresses auxin and efficiently regenerates doubled-haploid plants in Brassica napus
Source: BMC Plant Biol. 2012 Aug 2;12:127. doi: 10.1186/1471-2229-12-127 (PMC3464609; doi:10.1186/1471-2229-12-127)
Supplement: Additional file 2 — Statistical analysis of the contribution of several factors to embryo production in microspore cultures. 2 × 2 factor ANOVA for partitioning variance for microspore embryogenesis response of buds collected from donor plants grown in two different conditions and after isolation, subjected to two different thermal stress conditions (8 replicate data). (PDF 67 kb) [file 1471-2229-12-127-S2.pdf]

Additional file 2

Statistical analysis of the contribution of several factors to embryo production in microspore cultures

2×2 factor ANOVA for partitioning variance for microspore embryogenesis response of buds collected from donor plants grown in two different conditions and after isolation, subjected to two different thermal stress conditions (8 replicate data)

Source	Degree of freedom	Mean Squares
Growth conditions (GC)	1	2464755.0*
Thermal pretreatment (TP)	1	3703961.5*
Interaction (GC × TP)	1	1384032.0*
Error	28	49997.37

^{ns}– Non- significant at α=0.05

*- significant at α=0.05

Base experiment planned and executed as 2×3 factorial CRD (two donor growth conditions vis-à-vis 15°C day/ 10 °C night with 16 h photoperiod in growth chamber and 18 °C continues with ambient light conditions in green house and three thermal pre-treatments vis-à-vis 32°C, 18°C and 25°C after microspore isolation). However, since no embryogenesis was observed at 25°C pre-treatment, it was excluded from statistical analysis.
