# Supplementary material for: A new microspore embryogenesis system under low temperature which mimics zygotic embryogenesis initials, expresses auxin and efficiently regenerates doubled-haploid plants in Brassica napus
Source: BMC Plant Biol. 2012 Aug 2;12:127. doi: 10.1186/1471-2229-12-127 (PMC3464609; doi:10.1186/1471-2229-12-127)
Supplement: Additional file 3 — Statistical analysis of the contribution of several factors to microspore embryo germination. 2 × 2 × 3 factorial ANOVA for partitioning variation in response of embryo conversion to plantlets (germination) for microspore embryos obtained from two different pathways and from donor plants of growth chambers, cultured directly or air-desiccated and subjected to three different germination pre-treatments. (4 replicate each with 7–20 embryos cultured per replicate for each factor). (PDF 39 kb) [file 1471-2229-12-127-S3.pdf]

<u>Additional file 3</u>

Statistical analysis of the contribution of several factors to microspore embryo germination

2×2×3 factorial ANOVA for partitioning variation in response of embryo conversion to plantlets (germination) for microspore embryos obtained from two different pathway, cultured directly or air-desiccated and subjected to three different germination pre-treatments. (4 replicate each with 7-20 embryos cultured per replicate for each factor)

Source	Degree of freedom	Mean Squares
Embryo induction temperature (EIT)	1	38.9^{ns}
Desiccation treatment (DT)	1	7267.6*
Germination temperature treatment (GT)	2	3149.4*
Interaction		
EIT × DT	1	12.7^{ns}
EIT × GT	2	53.9^{ns}
DT × GT	2	1405.9*
EIT × DT × GT	2	3.6^{ns}
Error	36	35.5

^{ns}– Non- significant at α=0.05 *- significant at α=0.05
